# 1-[4-(Prop-2-en-1-yl­oxy)benz­yl]-2-[4-(prop-2-en-1-yl­oxy)phen­yl]-1*H*-benzimidazole

**DOI:** 10.1107/S160053681204559X

**Published:** 2012-11-10

**Authors:** Md. Lutfor Rahman, Huey Chong Kwong, Mashitah Mohd. Yusoff, Gurumurthy Hegde, Mohamed Ibrahim Mohamed Tahir

**Affiliations:** a University Malaysia Pahang, Faculty of Industrial Sciences and Technology, 26300 Gambang, Kuantan, Pahang, Malaysia; b Department Chemistry, Faculty Science, Universiti Putra Malaysia, 43400 UPM Serdang, Selangor, Malaysia

## Abstract

In the title compound, C_26_H_24_N_2_O_2_, the benzimidazole ring system is almost planar [maximum displacement = 0.025 (1) Å] and makes dihedral angles of 80.48 (5) and 41.57 (5)° with the benzene rings, which are inclined to one another by 65.33 (6)°. In the crystal, mol­ecules are linked *via* C—H⋯π and weak π–π inter­actions [centroid–centroid distance = 3.8070 (7) Å and inter­planar distance = 3.6160 (5) Å].

## Related literature

For the activity of benzimidazole derivatives against viruses, see: Tamm & Sehgal (1978 ▶); Porcari *et al.* (1998 ▶); Migawa *et al.* (1998 ▶). For their other biological activity, see: Spasov *et al.* (1999 ▶); Nakano *et al.* (2000 ▶); Zhao *et al.* (2000 ▶); White *et al.* (2000 ▶); Xiangming *et al.* (2007 ▶). For related structures, see: Kia *et al.* (2009 ▶); Zhou *et al.* (2009 ▶). For synthetic details, see: Lutfor *et al.* (2008 ▶). For standard bond lengths, see Allen *et al.* (1987 ▶).
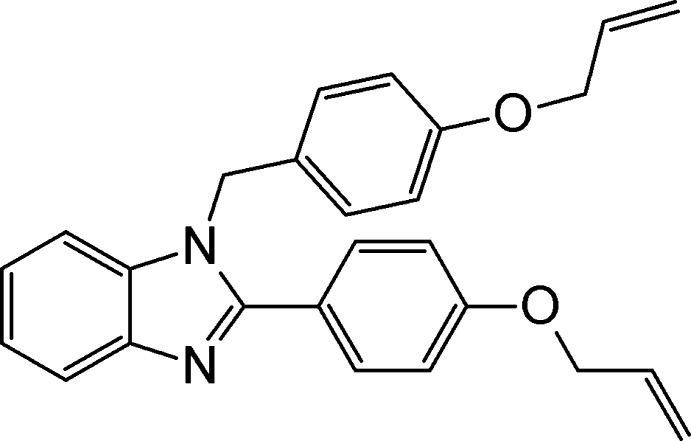


## Experimental

### 

#### Crystal data

C_26_H_24_N_2_O_2_
*M* *_r_* = 396.49
Monoclinic, 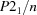

*a* = 12.5455 (1) Å
*b* = 10.1989 (1) Å
*c* = 15.9340 (2) Å
β = 99.5027 (11)°
*V* = 2010.78 (4) Å^3^
*Z* = 4
Cu *K*α radiation
μ = 0.66 mm^−1^
*T* = 100 K
0.35 × 0.23 × 0.08 mm

#### Data collection

Agilent Technologies Gemini diffractometer
Absorption correction: multi-scan (*CrysAlis PRO*; Agilent, 2011 ▶) *T*_min_ = 0.80, *T*_max_ = 0.95
38976 measured reflections
3908 independent reflections
3563 reflections with *I* > 2.0σ(*I*)
*R*_int_ = 0.029

#### Refinement

*R*[*F* ^2^ > 2σ(*F* ^2^)] = 0.038
*wR*(*F* ^2^) = 0.100
*S* = 0.99
3891 reflections
271 parameters
H-atom parameters constrained
Δρ_max_ = 0.26 e Å^−3^
Δρ_min_ = −0.25 e Å^−3^

### 

Data collection: *CrysAlis PRO* (Agilent, 2011 ▶); cell refinement: *CrysAlis PRO*; data reduction: *CrysAlis PRO*; program(s) used to solve structure: *SIR92* (Altomare *et al.*, 1994 ▶); program(s) used to refine structure: *CRYSTALS* (Betteridge *et al.*, 2003 ▶); molecular graphics: *Mercury* (Macrae *et al.*, 2008 ▶); software used to prepare material for publication: *CRYSTALS*.

## Supplementary Material

Crystal structure: contains datablock(s) global, I. DOI: 10.1107/S160053681204559X/su2508sup1.cif

Structure factors: contains datablock(s) I. DOI: 10.1107/S160053681204559X/su2508Isup2.hkl

Supplementary material file. DOI: 10.1107/S160053681204559X/su2508Isup3.cml

Additional supplementary materials:  crystallographic information; 3D view; checkCIF report

## Figures and Tables

**Table 1:** Hydrogen-bond geometry (Å, °) *Cg*2 is the centroid of the C2–C5/C26/C27 ring; *Cg*3 is the centroid of the C10–C15 ring and *Cg*4 is the centroid of the C16–C19/C24/25 ring.

*D*—H⋯*A*	*D*—H	H⋯*A*	*D*⋯*A*	*D*—H⋯*A*
C12—H121⋯*Cg*4^i^	0.97	2.62	3.5064 (13)	153
C23—H231⋯*Cg*3^ii^	0.96	2.73	3.6859 (15)	175
C30—H301⋯*Cg*2^iii^	0.95	2.72	3.6202 (14)	159

Symmetry codes: (i) 
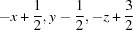
; (ii) 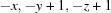; (iii) 
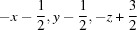
.

**Fig. 1.**
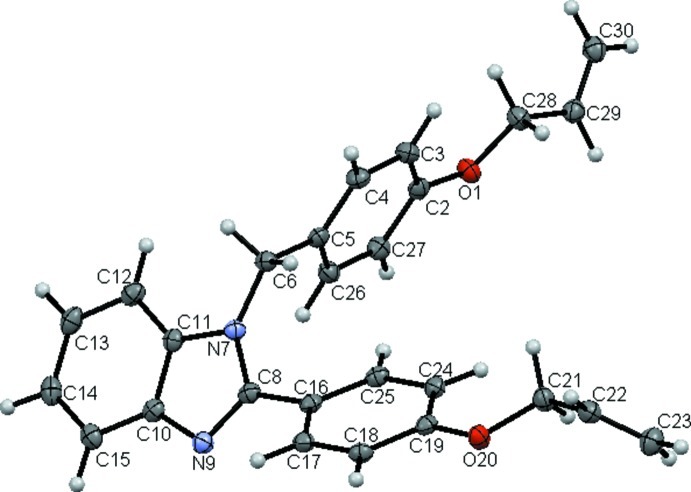
The molecular structure of the title molecule with the atom numbering. Displacement ellipsoids are drawn at the 50% probability level.

## supplementary crystallographic information

### Comment

The importance of the benzimidazole nucleus is due to the fact that it is found in many biologically active compounds (Xiangming *et al.*, 2007). Benzimidazole derivatives have been shown to have significant activity against several viruses such as, HIV (Porcari *et al.*, 1998), herpes (Migawa *et al.*, 1998), influenza (Tamm and Sehgal, 1978) and human cytomegalovirus (Porcari *et al.*, 1998). Many benzimidazole containing compounds exhibited significant biological activities, such as novel anti-allergic agents (Nakano *et al.*, 2000), factor Xa inhibitors (Zhao *et al.*, 2000), poly (ADP-ribose) polymerase (PARP) inhibitors (White *et al.*, 2000). Some substituted benzimidazole derivatives have been recently commercialized for applications in veterinarian medicine, and in diverse human therapeutic areas such as, the treatment of ulcers and as antihistamines (Spasov *et al.*, 1999).

In the titled compound (Fig. 1) the bond lengths (Allen *et al.*, 1987) and bond angles are normal. The benzimidazole ring system (N7/C8/N9/C10-C15) is almost planar, with a maximum displacement of 0.025 (1) Å for atom C8. The dihedral angle formed by the mean plane of the benzimidazole ring system and the two adjacent benzene rings (C2-C5/C26/C27 and C16-C19/C24/C25) are 80.48 (5)° and 41.57 (5)°, respectively. The benzene rings themselves are inclined to one another by 65.33 (6)°, rather than ca. 90° as observed elsewhere, for example in the related compounds, 4-[1-(4-Cyanobenzyl)-1*H*-benzimidazol-2-yl]benzonitrile (Kia *et al.*, 2009) and 1-(4-tert-Butylbenzyl)-2-(4-tert-butylphenyl)-1H-benzimidazole (Zhou *et al.*, 2009). The torsion angles of the allyloxy chains (O1-C28-C29-C30 and O20-C21-C22-C23) are 133.16 (13)° and 120.11 (14)°, respectively.

In the crystal, molecules are linked via C-H···π interactions (Table 1) and weak π-π interactions. The centroid to centroid distance, *Cg*1···*Cg*2^i^, is 3.8070 (7)Å with an interplanar distance of 3.6160 (5) Å [*Cg*1 and *Cg*2 are the centroids of the N7/C8/N9/C10/C11 and C2-C5/C26/C27 rings; symmetry code: (i) -x + 1/2, y + 1/2, -z + 3/2].

### Experimental

4-Hydroxybenzaldehyde (10.0 g, 83.25 mmol) was dissolved in dry acetone (150 mL). Then allyl bromide (12.097 g, 100 mmol), potassium carbonate (13.80 g, 100 mmol) and a catalytic amount of potassium iodide (20 mg) were added and the mixture was refluxed for 24 h under argon atmosphere. Afterwards, it was poured into ice-cold water and acidified with dilute hydrochloric acid (pH<5). The precipitate was filtered off and was crystallized from methanol/chloroform to yield the 4-(prop-2-en-1-yloxy)benzaldehyde. A solution of 4-(prop-2-en-1-yloxy)benzaldehyde (5.00 g, 30.82 mmol) in ethanol was added to a solution of *o*-phenylenediamine (1.66 g, 15.41 mmol) in ethanol. The mixture was refluxed with a few drops of acetic acid as catalyst for 12 h to yield the title compound as a slightly grey solid. The product was filtered off and recrystallized from absolute ethanol to give pale-brown block-like crystals of the title compound, suitable for X-ray diffraction analysis (Lutfor *et al.*, 2008).

### Refinement

The H atoms were all located in a difference Fourier map, but those attached to carbon atoms were repositioned geometrically. The H atoms were initially refined with soft restraints on the bond lengths and angles to regularize their geometry (C—H in the range 0.93–0.98 Å, N—H in the range 0.86–0.90 Å) and *U*_iso_(H) in the range 1.2–1.5 times *U*_eq_ of the parent atom). In the final cycles of refinement they were refined as riding atoms.

### Crystal data

**Table:**

C_26_H_24_N_2_O_2_	*F*(000) = 840
*M**_r_* = 396.49	*D*_x_ = 1.310 Mg m^−^^3^
Monoclinic, *P*2_1_/*n*	Cu *K*α radiation, λ = 1.54180 Å
Hall symbol: -P 2yn	Cell parameters from 17352 reflections
*a* = 12.5455 (1) Å	θ = 4–71°
*b* = 10.1989 (1) Å	µ = 0.66 mm^−^^1^
*c* = 15.9340 (2) Å	*T* = 100 K
β = 99.5027 (11)°	Block, pale brown
*V* = 2010.78 (4) Å^3^	0.35 × 0.23 × 0.08 mm
*Z* = 4	

### Data collection

**Table:**

Agilent Technologies Gemini diffractometer	3908 independent reflections
Radiation source: sealed x-ray tube	3563 reflections with *I* > 2.0σ(*I*)
Graphite monochromator	*R*_int_ = 0.029
ω scans	θ_max_ = 71.4°, θ_min_ = 4.2°
Absorption correction: multi-scan (*CrysAlis PRO*; Agilent, 2011)	*h* = −15→15
*T*_min_ = 0.80, *T*_max_ = 0.95	*k* = −12→12
38976 measured reflections	*l* = −19→19

### Refinement

**Table:**

Refinement on *F*^2^	Primary atom site location: structure-invariant direct methods
Least-squares matrix: full	Hydrogen site location: difference Fourier map
*R*[*F*^2^ > 2σ(*F*^2^)] = 0.038	H-atom parameters constrained
*wR*(*F*^2^) = 0.100	Method = Modified Sheldrick *w* = 1/[σ^2^(*F*^2^) + (0.06*P*)^2^ + 1.04*P*], where *P* = (max(*F*_o_^2^,0) + 2*F*_c_^2^)/3
*S* = 0.99	(Δ/σ)_max_ = 0.002
3891 reflections	Δρ_max_ = 0.26 e Å^−^^3^
271 parameters	Δρ_min_ = −0.25 e Å^−^^3^
0 restraints	

### Special details

**Table:**

Geometry. Bond distances, angles etc. have been calculated using the rounded fractional coordinates. All su's are estimated from the variances of the (full) variance-covariance matrix. The cell esds are taken into account in the estimation of distances, angles and torsion angles

### Fractional atomic coordinates and isotropic or equivalent isotropic displacement parameters (Å^2^)

**Table:**

	*x*	*y*	*z*	*U*_iso_*/*U*_eq_	
O1	−0.10938 (7)	0.03132 (9)	0.75160 (6)	0.0210 (3)	
O20	−0.08975 (7)	0.86587 (8)	0.53875 (6)	0.0204 (3)	
N7	0.26914 (8)	0.41707 (10)	0.66850 (6)	0.0168 (3)	
N9	0.32584 (8)	0.49128 (10)	0.55014 (7)	0.0187 (3)	
C2	−0.02893 (10)	0.12293 (12)	0.75473 (8)	0.0183 (3)	
C3	−0.01085 (10)	0.22357 (13)	0.81468 (8)	0.0207 (3)	
C4	0.06903 (10)	0.31589 (13)	0.80889 (8)	0.0205 (3)	
C5	0.13284 (9)	0.30980 (12)	0.74543 (7)	0.0174 (3)	
C6	0.21706 (10)	0.41599 (12)	0.74388 (7)	0.0180 (3)	
C8	0.25200 (10)	0.50155 (12)	0.59986 (8)	0.0169 (3)	
C10	0.39729 (10)	0.39581 (12)	0.58841 (8)	0.0180 (3)	
C11	0.36292 (9)	0.34893 (12)	0.66226 (8)	0.0174 (3)	
C12	0.41964 (10)	0.25513 (12)	0.71540 (8)	0.0207 (3)	
C13	0.51416 (10)	0.20871 (13)	0.69185 (9)	0.0233 (4)	
C14	0.55011 (10)	0.25460 (13)	0.61838 (9)	0.0227 (4)	
C15	0.49311 (10)	0.34782 (12)	0.56574 (8)	0.0208 (3)	
C16	0.16006 (10)	0.59333 (12)	0.58484 (7)	0.0170 (3)	
C17	0.17631 (10)	0.72115 (12)	0.55660 (7)	0.0181 (3)	
C18	0.09167 (10)	0.80926 (12)	0.54134 (8)	0.0185 (3)	
C19	−0.01170 (10)	0.77117 (12)	0.55381 (7)	0.0172 (3)	
C21	−0.19187 (10)	0.83706 (13)	0.56480 (8)	0.0218 (4)	
C22	−0.25815 (10)	0.95958 (13)	0.55465 (8)	0.0211 (3)	
C23	−0.35254 (11)	0.96766 (14)	0.50457 (9)	0.0262 (4)	
C24	−0.02971 (10)	0.64406 (12)	0.58006 (8)	0.0181 (3)	
C25	0.05619 (10)	0.55642 (12)	0.59561 (7)	0.0180 (3)	
C26	0.11566 (10)	0.20690 (12)	0.68709 (8)	0.0190 (3)	
C27	0.03538 (10)	0.11448 (12)	0.69133 (8)	0.0196 (3)	
C28	−0.18801 (10)	0.05439 (13)	0.80606 (8)	0.0212 (3)	
C29	−0.27537 (10)	−0.04607 (13)	0.78754 (8)	0.0208 (3)	
C30	−0.31212 (10)	−0.11109 (13)	0.84837 (9)	0.0229 (4)	
H31	−0.05350	0.22920	0.86000	0.0269*	
H41	0.08040	0.38680	0.84960	0.0251*	
H61	0.27430	0.40430	0.79340	0.0235*	
H62	0.18260	0.50240	0.74740	0.0230*	
H121	0.39610	0.22590	0.76710	0.0255*	
H131	0.55580	0.14310	0.72760	0.0285*	
H141	0.61750	0.22060	0.60530	0.0280*	
H151	0.51880	0.37840	0.51560	0.0261*	
H171	0.24900	0.74770	0.54950	0.0226*	
H181	0.10260	0.89680	0.52220	0.0235*	
H211	−0.22950	0.76570	0.52890	0.0264*	
H212	−0.17880	0.80930	0.62670	0.0282*	
H221	−0.22850	1.03690	0.58740	0.0259*	
H231	−0.38010	0.89420	0.46990	0.0337*	
H232	−0.39470	1.04670	0.50120	0.0328*	
H241	−0.10180	0.61580	0.58640	0.0238*	
H251	0.04310	0.46810	0.61320	0.0223*	
H261	0.15810	0.20230	0.64160	0.0234*	
H271	0.02280	0.04320	0.65050	0.0239*	
H281	−0.22060	0.14390	0.79440	0.0266*	
H282	−0.15390	0.04870	0.86720	0.0255*	
H291	−0.30710	−0.06000	0.72780	0.0250*	
H301	−0.36980	−0.17230	0.83560	0.0284*	
H302	−0.27840	−0.09670	0.90660	0.0289*	

### Atomic displacement parameters (Å^2^)

**Table:**

	*U*^11^	*U*^22^	*U*^33^	*U*^12^	*U*^13^	*U*^23^
O1	0.0197 (4)	0.0206 (4)	0.0237 (5)	−0.0026 (3)	0.0067 (4)	−0.0017 (4)
O20	0.0186 (4)	0.0183 (4)	0.0248 (5)	0.0026 (3)	0.0054 (3)	0.0036 (3)
N7	0.0171 (5)	0.0166 (5)	0.0167 (5)	0.0001 (4)	0.0027 (4)	0.0015 (4)
N9	0.0188 (5)	0.0188 (5)	0.0189 (5)	0.0005 (4)	0.0040 (4)	0.0009 (4)
C2	0.0165 (6)	0.0176 (6)	0.0202 (6)	0.0009 (5)	0.0010 (5)	0.0044 (5)
C3	0.0208 (6)	0.0250 (6)	0.0170 (6)	−0.0007 (5)	0.0048 (5)	0.0012 (5)
C4	0.0225 (6)	0.0224 (6)	0.0163 (6)	−0.0005 (5)	0.0020 (5)	−0.0018 (5)
C5	0.0167 (6)	0.0184 (6)	0.0164 (6)	0.0022 (5)	0.0010 (4)	0.0040 (5)
C6	0.0198 (6)	0.0193 (6)	0.0149 (6)	−0.0002 (5)	0.0029 (5)	0.0009 (5)
C8	0.0193 (6)	0.0153 (6)	0.0155 (6)	−0.0022 (5)	0.0010 (5)	−0.0004 (4)
C10	0.0190 (6)	0.0155 (6)	0.0190 (6)	−0.0020 (5)	0.0014 (5)	−0.0011 (5)
C11	0.0166 (6)	0.0158 (6)	0.0193 (6)	−0.0017 (5)	0.0012 (5)	−0.0020 (5)
C12	0.0217 (6)	0.0179 (6)	0.0211 (6)	−0.0014 (5)	−0.0002 (5)	0.0018 (5)
C13	0.0216 (6)	0.0182 (6)	0.0276 (7)	0.0019 (5)	−0.0032 (5)	0.0004 (5)
C14	0.0162 (6)	0.0211 (6)	0.0302 (7)	0.0003 (5)	0.0023 (5)	−0.0062 (5)
C15	0.0200 (6)	0.0194 (6)	0.0234 (6)	−0.0025 (5)	0.0051 (5)	−0.0034 (5)
C16	0.0201 (6)	0.0178 (6)	0.0126 (5)	0.0009 (5)	0.0012 (4)	−0.0012 (4)
C17	0.0190 (6)	0.0200 (6)	0.0155 (6)	−0.0011 (5)	0.0035 (4)	0.0000 (5)
C18	0.0229 (6)	0.0157 (6)	0.0170 (6)	−0.0006 (5)	0.0035 (5)	0.0015 (5)
C19	0.0194 (6)	0.0180 (6)	0.0137 (5)	0.0022 (5)	0.0011 (4)	−0.0012 (4)
C21	0.0176 (6)	0.0221 (6)	0.0262 (7)	0.0002 (5)	0.0049 (5)	0.0028 (5)
C22	0.0222 (6)	0.0210 (6)	0.0209 (6)	0.0004 (5)	0.0063 (5)	0.0009 (5)
C23	0.0242 (7)	0.0251 (7)	0.0285 (7)	0.0027 (5)	0.0019 (5)	−0.0005 (5)
C24	0.0171 (6)	0.0194 (6)	0.0173 (6)	−0.0013 (5)	0.0015 (4)	−0.0007 (5)
C25	0.0220 (6)	0.0154 (6)	0.0158 (6)	−0.0014 (5)	0.0008 (5)	−0.0005 (4)
C26	0.0187 (6)	0.0205 (6)	0.0185 (6)	0.0023 (5)	0.0051 (5)	0.0014 (5)
C27	0.0205 (6)	0.0179 (6)	0.0202 (6)	0.0019 (5)	0.0030 (5)	−0.0019 (5)
C28	0.0200 (6)	0.0223 (6)	0.0223 (6)	0.0003 (5)	0.0068 (5)	−0.0001 (5)
C29	0.0187 (6)	0.0228 (6)	0.0205 (6)	0.0015 (5)	0.0023 (5)	−0.0016 (5)
C30	0.0207 (6)	0.0230 (6)	0.0251 (7)	−0.0018 (5)	0.0040 (5)	−0.0030 (5)

### Geometric parameters (Å, º)

**Table:**

O1—C2	1.3702 (15)	C24—C25	1.3905 (18)
O1—C28	1.4367 (16)	C26—C27	1.3892 (18)
O20—C19	1.3681 (15)	C28—C29	1.4937 (18)
O20—C21	1.4407 (16)	C29—C30	1.3188 (19)
N7—C6	1.4590 (15)	C3—H31	0.9700
N7—C8	1.3810 (16)	C4—H41	0.9700
N7—C11	1.3841 (15)	C6—H61	0.9800
N9—C8	1.3187 (16)	C6—H62	0.9900
N9—C10	1.3939 (16)	C12—H121	0.9700
C2—C3	1.3950 (18)	C13—H131	0.9700
C2—C27	1.3957 (18)	C14—H141	0.9700
C3—C4	1.3892 (18)	C15—H151	0.9600
C4—C5	1.3908 (17)	C17—H171	0.9800
C5—C6	1.5161 (17)	C18—H181	0.9600
C5—C26	1.3949 (17)	C21—H211	1.0000
C8—C16	1.4740 (18)	C21—H212	1.0100
C10—C11	1.4024 (18)	C22—H221	0.9800
C10—C15	1.3995 (18)	C23—H231	0.9600
C11—C12	1.3927 (17)	C23—H232	0.9600
C12—C13	1.3849 (18)	C24—H241	0.9700
C13—C14	1.4026 (19)	C25—H251	0.9700
C14—C15	1.3859 (18)	C26—H261	0.9700
C16—C17	1.4049 (17)	C27—H271	0.9700
C16—C25	1.3939 (18)	C28—H281	1.0000
C17—C18	1.3816 (18)	C28—H282	1.0000
C18—C19	1.3990 (18)	C29—H291	0.9800
C19—C24	1.3921 (17)	C30—H301	0.9500
C21—C22	1.4949 (19)	C30—H302	0.9700
C22—C23	1.3167 (19)		
			
C2—O1—C28	116.19 (10)	C5—C4—H41	119.00
C19—O20—C21	116.76 (9)	N7—C6—H61	107.00
C6—N7—C8	128.63 (10)	N7—C6—H62	107.00
C6—N7—C11	123.84 (10)	C5—C6—H61	109.00
C8—N7—C11	106.23 (10)	C5—C6—H62	109.00
C8—N9—C10	104.92 (10)	H61—C6—H62	109.00
O1—C2—C3	124.21 (11)	C11—C12—H121	122.00
O1—C2—C27	116.22 (11)	C13—C12—H121	121.00
C3—C2—C27	119.54 (11)	C12—C13—H131	119.00
C2—C3—C4	119.39 (12)	C14—C13—H131	120.00
C3—C4—C5	121.74 (12)	C13—C14—H141	118.00
C4—C5—C6	117.79 (11)	C15—C14—H141	120.00
C4—C5—C26	118.29 (11)	C10—C15—H151	121.00
C6—C5—C26	123.92 (10)	C14—C15—H151	121.00
N7—C6—C5	115.27 (10)	C16—C17—H171	119.00
N7—C8—N9	113.16 (11)	C18—C17—H171	120.00
N7—C8—C16	122.69 (11)	C17—C18—H181	121.00
N9—C8—C16	124.15 (11)	C19—C18—H181	119.00
N9—C10—C11	109.92 (11)	O20—C21—H211	110.00
N9—C10—C15	130.48 (11)	O20—C21—H212	109.00
C11—C10—C15	119.57 (11)	C22—C21—H211	110.00
N7—C11—C10	105.77 (10)	C22—C21—H212	110.00
N7—C11—C12	131.02 (11)	H211—C21—H212	110.00
C10—C11—C12	123.18 (11)	C21—C22—H221	117.00
C11—C12—C13	116.39 (12)	C23—C22—H221	120.00
C12—C13—C14	121.32 (12)	C22—C23—H231	120.00
C13—C14—C15	121.95 (12)	C22—C23—H232	121.00
C10—C15—C14	117.59 (12)	H231—C23—H232	119.00
C8—C16—C17	119.36 (11)	C19—C24—H241	121.00
C8—C16—C25	122.07 (11)	C25—C24—H241	120.00
C17—C16—C25	118.56 (11)	C16—C25—H251	120.00
C16—C17—C18	120.79 (12)	C24—C25—H251	119.00
C17—C18—C19	119.86 (11)	C5—C26—H261	119.00
O20—C19—C18	115.62 (11)	C27—C26—H261	120.00
O20—C19—C24	124.28 (11)	C2—C27—H271	119.00
C18—C19—C24	120.10 (11)	C26—C27—H271	121.00
O20—C21—C22	107.67 (10)	O1—C28—H281	110.00
C21—C22—C23	123.12 (12)	O1—C28—H282	111.00
C19—C24—C25	119.52 (12)	C29—C28—H281	109.00
C16—C25—C24	121.15 (11)	C29—C28—H282	110.00
C5—C26—C27	120.77 (11)	H281—C28—H282	109.00
C2—C27—C26	120.24 (11)	C28—C29—H291	117.00
O1—C28—C29	108.84 (10)	C30—C29—H291	120.00
C28—C29—C30	122.26 (12)	C29—C30—H301	121.00
C2—C3—H31	120.00	C29—C30—H302	119.00
C4—C3—H31	120.00	H301—C30—H302	120.00
C3—C4—H41	119.00		
			
C28—O1—C2—C3	9.44 (17)	C4—C5—C26—C27	1.28 (18)
C28—O1—C2—C27	−168.51 (11)	N9—C8—C16—C17	−40.64 (17)
C2—O1—C28—C29	173.88 (10)	N7—C8—C16—C25	−42.45 (17)
C21—O20—C19—C18	−169.69 (10)	N7—C8—C16—C17	139.28 (12)
C21—O20—C19—C24	10.16 (16)	N9—C8—C16—C25	137.63 (13)
C19—O20—C21—C22	171.61 (10)	N9—C10—C15—C14	177.71 (13)
C6—N7—C11—C10	−168.56 (11)	C11—C10—C15—C14	0.06 (19)
C8—N7—C11—C10	−0.61 (13)	C15—C10—C11—C12	−0.17 (19)
C6—N7—C11—C12	9.7 (2)	N9—C10—C11—N7	0.11 (14)
C8—N7—C11—C12	177.59 (13)	N9—C10—C11—C12	−178.27 (11)
C8—N7—C6—C5	104.28 (14)	C15—C10—C11—N7	178.21 (11)
C11—N7—C6—C5	−90.59 (14)	N7—C11—C12—C13	−177.85 (13)
C6—N7—C8—N9	168.14 (11)	C10—C11—C12—C13	0.08 (19)
C11—N7—C8—N9	0.97 (14)	C11—C12—C13—C14	0.11 (19)
C6—N7—C8—C16	−11.79 (19)	C12—C13—C14—C15	−0.2 (2)
C11—N7—C8—C16	−178.95 (11)	C13—C14—C15—C10	0.1 (2)
C10—N9—C8—N7	−0.88 (14)	C8—C16—C17—C18	179.53 (11)
C10—N9—C8—C16	179.04 (11)	C25—C16—C17—C18	1.20 (17)
C8—N9—C10—C11	0.46 (14)	C8—C16—C25—C24	−179.18 (11)
C8—N9—C10—C15	−177.37 (13)	C17—C16—C25—C24	−0.90 (17)
O1—C2—C3—C4	−176.16 (12)	C16—C17—C18—C19	−0.19 (18)
O1—C2—C27—C26	176.99 (11)	C17—C18—C19—O20	178.71 (11)
C27—C2—C3—C4	1.74 (19)	C17—C18—C19—C24	−1.16 (18)
C3—C2—C27—C26	−1.07 (19)	O20—C19—C24—C25	−178.39 (11)
C2—C3—C4—C5	−0.92 (19)	C18—C19—C24—C25	1.46 (18)
C3—C4—C5—C6	179.34 (11)	O20—C21—C22—C23	120.11 (14)
C3—C4—C5—C26	−0.59 (19)	C19—C24—C25—C16	−0.42 (18)
C4—C5—C6—N7	−170.42 (11)	C5—C26—C27—C2	−0.46 (19)
C6—C5—C26—C27	−178.65 (11)	O1—C28—C29—C30	133.16 (13)
C26—C5—C6—N7	9.50 (17)		

### Hydrogen-bond geometry (Å, º)

Cg2 is the centroid of the C2–C5/C26/C27 ring; Cg3 is the centroid of the C10–C15 ring and Cg4 is the centroid of the C16–C19/C24/25 ring.

**Table:**

*D*—H···*A*	*D*—H	H···*A*	*D*···*A*	*D*—H···*A*
C12—H121···*Cg*4^i^	0.97	2.62	3.5064 (13)	153
C23—H231···*Cg*3^ii^	0.96	2.73	3.6859 (15)	175
C30—H301···*Cg*2^iii^	0.95	2.72	3.6202 (14)	159

Symmetry codes: (i) −*x*+1/2, *y*−1/2, −*z*+3/2; (ii) −*x*, −*y*+1, −*z*+1; (iii) −*x*−1/2, *y*−1/2, −*z*+3/2.
